# Arthroscopic-assisted reduction, bone grafting and screw fixation across the scapholunate joint for proximal pole scaphoid nonunion

**DOI:** 10.1186/s12891-020-03850-w

**Published:** 2020-12-10

**Authors:** Jung-Pan Wang, Hui-Kuang Huang, Jui-Tien Shih

**Affiliations:** 1 Department of Surgery, School of Medicine, National Yang-Ming University, Taipei, Taiwan; 2 Department of Orthopaedics & Traumatology, Taipei Veterans General Hospital, Taipei, Taiwan; 3 Department of Orthopaedics, Chiayi Christian Hospital, Chiayi, Taiwan; 4 Chung Hwa University of Medical Technology, Tainan, Taiwan; 5 Department of Orthopaedic Surgery, Taoyuan Armed Forces General Hospital, No. 168 Jong-Shing Road, Taoyuan County, Taiwan

**Keywords:** Arthroscopy, Bone transplantation, Fractures, Scaphoid bone, Ununited

## Abstract

**Background:**

There are some difficulties in treating proximal scaphoid nonunion, mainly including poor vascularity of the proximal scaphoid fragment and limited space for a stable fixation in the proximal scaphoid fragment. This study reports the outcomes of treating proximal scaphoid nonunion with arthroscopic assist for reduction, bone grafting and screw fixation across the scapholunate (SL) joint.

**Methods:**

Between 2008 and 2017, 21 patients were enrolled. Fracture healing and change in the lateral SL angle and SL gap were evaluated. Functional outcomes were evaluated using the Disabilities of the Arm, Shoulder, and Hand (DASH) score, wrist range of motion, grip strength, and the Visual Analog Scale (VAS) for pain.

**Results:**

Nineteen patients achieved fracture healing and their mean follow-up duration was 31.3 months (24–120 months). The average fracture healing time was 16.3 weeks (10 to 28 weeks). From the preoperative to the postoperative final evaluation, there was some significant improvement in wrist function, including wrist flexion from 54.5^o^ to 67.4^o^, wrist extension from 62.3^o^ to 71.7^o^, DASH scores from 52.4 to 21.4, VAS during activity from 4.6 to 2.1, and grip strength from 9.6 kg to 24.7 kg. The lateral SL angle also improved significantly, from 82^o^ to 66^o^. Seventeen patients requested screw removal after fracture healing because of their cultural belief in not leaving hardware in the body. No significant SL gap widening was noted after screw removal in the sequential follow-ups.

**Conclusions:**

Using arthroscopic-assisted reduction, bone grafting and screw fixation across the SL joint in proximal scaphoid nonunion treatment, satisfactory functional and radiographic outcomes can be achieved.

## Background

The scaphoid has a unique intraosseous retrograde blood flow, and it connects with the lunate in the proximal row to sustain axial loading of the wrist [1]. From 5 to 15% of all scaphoid fractures develop to nonunion [2, 3]. Scaphoid nonunion can cause changes in wrist mechanics and uncoupling of carpal rows, eventually progressing to scaphoid nonunion advanced collapse (SNAC) [4]. SNAC change was found in 97% of patients with at least 5 years of scaphoid nonunion [5]. Treatment for scaphoid nonunion includes non-vascularized bone grafts, pedicle vascularized grafts, free flaps, and total arthroscopic management. The majority of reports for each method indicates high union rates of more than 90%, but there is still debate regarding which treatment is better for patients [2, 3, 6].

If a proximal scaphoid fracture failed to heal, there would be some important issues to be concerned about in the treatment, including poor vascularity of the proximal scaphoid fragment or avascular necrosis of the scaphoid, and limited space for stable fixation in the proximal fragment [7–9].

There have been several reports regarding treatment methods for proximal scaphoid nonunion. Both vascularized and non-vascularized bone graft combined with scaphoid screw or K-wires fixation are commonly used methods [10–16]. Precise and stable fixation of the small proximal fragment with the larger distal fragment is mostly achievable, but many surgeons still consider fixation in a small fragment to be a difficult procedure.

The reduction-association of the scapholunate joint (RASL) method has been proposed as a treatment for chronic scapholunate (SL) interosseous ligament (SLIL) injury [17], using either an open or arthroscopic (ARASL) method with headless screw fixation across the SL joint after denuding the space between the scaphoid and the lunate [18]. Screw transfixation from the scaphoid to the lunate has also been reported as a way to overcome the difficulty of fixing the proximal scaphoid nonunion [9, 19].

Since arthroscopic treatment for scaphoid nonunion has become more commonly used, we designed this study to evaluate the outcomes of arthroscopic-assisted reduction, bone grafting and, using a small incision at the anatomic snuffbox region, screw fixation across the SL joint in treating proximal scaphoid nonunion.

## Methods

This study was approved by the ethics committee of our institution. We retrospectively evaluated cases of proximal scaphoid nonunion that were treated with arthroscopic-assisted bone grafting and headless compression screw fixation across the SL joint. All surgeries were performed by 2 hand surgeons (experience level III – experienced specialist) [20].

All patients underwent radiographic and magnetic resonance imaging (MRI) evaluation of their wrists preoperatively. We defined nonunion in our enrollment criteria as a persistently non-healed fracture 6 months after the trauma, based on radiographic findings. Inclusion criteria were (1) patients with a nonunion of a proximal scaphoid fracture, type I or type II, according to the Schernberg classification [21], (2) patients with no or only stage I SNAC [22], and (3) any age or gender.

Exclusion criteria were (1) stages II to IV SNAC or mid-carpal instability, and (2) patients without follow-up for 2 years or without complete medical records. Patients with more advanced SNAC may be suitable for proximal row carpectomy or some arthrodesis procedures. Patients with mid-carpal instability should undergo ligament tensioning procedures, and this may interfere with functional outcomes.

### Surgical technique

The surgical procedure was performed under general anesthesia and pneumatic tourniquet control with the forearm on the traction tower. The midcarpal portals were used for assessment and treatment of the scaphoid. Debridement of the nonunion site was performed with curette, burr, and shaver, until normal cancellous bone with punctate bleeding was observed.

If there was dorsal intercalated segmental instability (DISI), reduction of the lunate together with the fractured scaphoid proximal fragment was performed. Then, temporary transfixing of the radius and lunate was achieved by pinning from the dorsal aspect of the distal radius to the lunate.

If there was flexion and pronation of the distal scaphoid fragment, we would perform traction of the thumb and manually push on the scaphoid at the scaphoid tubercle, with force applied from palmar to dorsal. Then, temporary transfixing of the distal scaphoid fragment and the capitate or the lunate from the radial side of the scaphoid was performed to maintain the reduction hands-free. Our preference is to perform scaphocapitate transfixation. Positioning of these wires should be eccentric to avoid hindering the subsequent placement of the SL screw. It is important not to injure the surrounding vital tissues when setting the K-wires; using a small incision with instruments such as the Mosquito forceps for protection would be helpful.

We used the dry arthroscopy method when performing bone grafting. We harvested the autologous cancellous bone grafts (8 cases from the iliac crest and 13 from the distal radius) and cut them into small chips. The bone graft of the distal radius was harvested from the volar side through a small incision, about 2–3 cm in length. Then, we delivered the bone graft into the nonunion gap using a 2.9 mm arthroscopic cannula. A small freer elevator can be used to compress the bone graft to fill the bone defect.

**Fig. 1:**
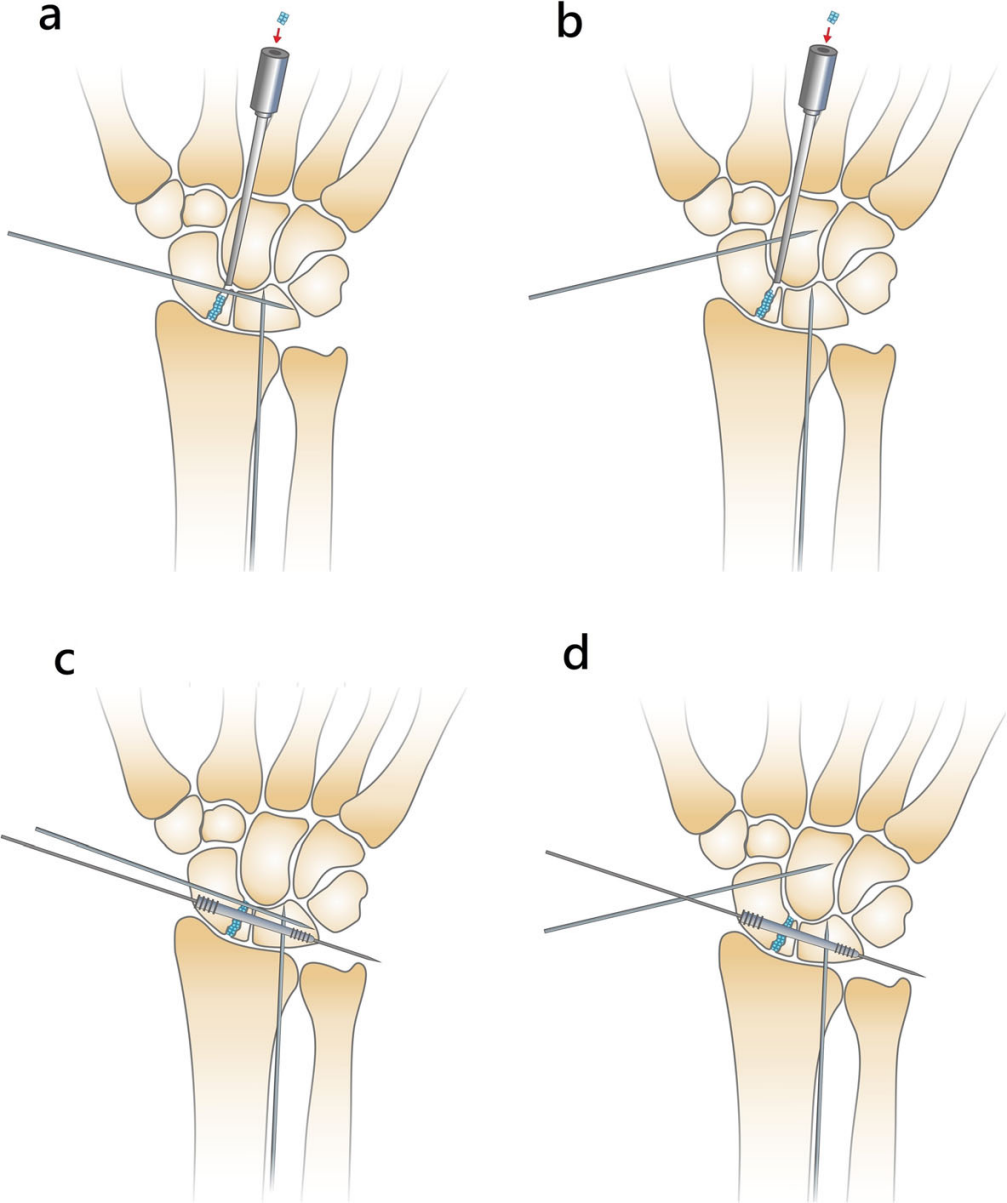
(**a**) and (**b**) After debridement and reduction of the fracture, the radiolunate and either the scapholunate or scaphocapitate joint are transfixed. The bone graft is set into the nonunion gap with arthroscopic assistance. (**c**) and (**d**) The headless compression screw for scapholunate transfixation

**Fig. 2:**
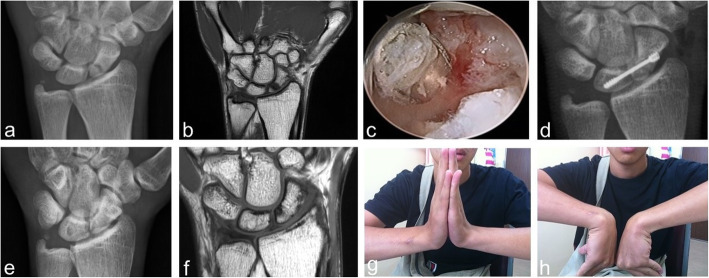
A 20-year-old male. (Case 17) (**a**) and (**b**) Radiograph and MRI 7 months after the fracture event showing the left proximal scaphoid nonunion. (**c**) Arthroscopic bone grafting. (**d**) Radiographs at the postoperative 1-month follow-up. (**e**) and (f) Radiograph and MRI at postoperative 7 years, revealing a solid union. (The screw was removed 8 months after surgery). (g) and (h) The patient regained good range of motion of the wrist

**Fig. 3:**
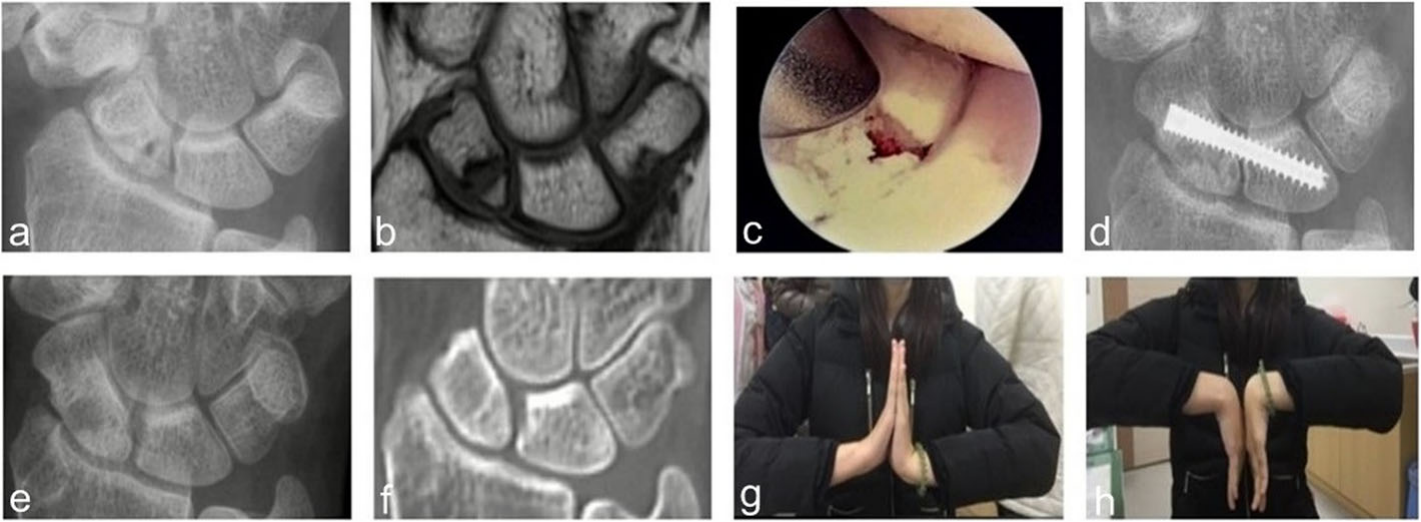
A 19-year-old female. (Case 18) (**a**) and (**b**) Radiograph and MRI 9 months after the fracture event showing the right scaphoid proximal pole nonunion. (**c**) Arthroscopic bone grafting. (**d**) Radiographs at the postoperative 1-month follow-up. (**e**) and (**f**) Radiograph and CT at postoperative 40 months, revealing a solid union. (The screw was removed 6 months after surgery). (**g**) and (h) The patient regained nearly full range of motion of the wrist

Then, a cannulated headless compression screw was introduced via a small incision at the anatomic snuffbox region to transfix the fracture site and cross the SL interval under fluoroscopic guidance [10] (Fig. 1). Either a Herbert screw, a 3.0 mm Headless Compression Screw (HCS) (DePuy Synthes, West Chester, PA, USA) (Fig. 2), or an Acutrak Standard (Acumed, OR, USA) screw was used for fixation (Fig. 3). Radial styloidectomy could be performed in the same, but more extended, screw insertion wound to gain better access or if the patients had symptoms of early-stage SNAC. Our incision for screw insertion is about 0.5 cm in length, and it can be extended to 1–2 cm proximally and volarly if the radial styloid is to be addressed. It is important that after setting the guide pin and screw, arthroscopic checking of the reduction and that the hardware has not penetrated the midcarpal joint should be performed. We did not aim to perform screw fixation through the center of the proximal scaphoid fragment. The goal was to have good headless screw fixation in the bigger part of the distal scaphoid fragment and the lunate, with the proximal fragment immobilized and fixed between them.

A removable short-arm thumb spica splint was applied postoperatively for 2 months, and then was intermittently used, depending on the condition of fracture healing and the patient’s rehabilitation. Rehabilitation programs with active gentle wrist range of motion exercise were initiated 2 months after surgery. Strengthening programs and a gradual return to unrestricted activities were allowed when union was radiographically confirmed.

### Outcome evaluation

After operation, patient follow-up was arranged routinely once every 2 weeks in the first month and once every month thereafter, until 3 months after the fracture had healed. Then, follow-up every 6 months was arranged until 2 years after the operation. After that, annual follow-up was recommended. Therefore, all our patients would have monthly radiographic evaluations for the first 6–7 months.

Radiographic examinations, including posteroanterior and lateral views of the wrist and posteroanterior and oblique views of the hand, were performed at each follow-up, except the first follow-up for removal of the stitches. Evaluation of the radiographs was carried out by 2 independent observers who were blinded to the results. Fracture healing, the lateral SL angle, and the SL gap were evaluated and measured. The lateral SL angle generally ranged from 30° to 60°, and a widening of the SL gap of more than 3 mm was considered suspicious of SL dissociation [23]. Computed tomography (CT) scan was not routinely performed, but only when fracture healing could not be confirmed. Functional outcomes, including wrist range of motion, the Visual Analog Scale (VAS) (0: no pain; 10: worst pain) during activity, and the Disabilities of the Arm, Shoulder, and Hand (DASH) score [24], were evaluated at each follow-up after fracture healing was noted.

All collected data were examined using the SPSS software package (version 22; SPSS, Chicago, IL, USA). Functional evaluations data were presented as continuous response variables and were examined using the paired 2-tailed Student’s t-test for identification of improvement. The *P*-value was set at 0.05 before analysis.

## Results

Twenty-one patients who underwent proximal scaphoid nonunion treatment from 2008 to 2017, and who met the inclusion criteria, were enrolled (Table 1). The mean age was 31.8 years (range, 19–55 years). The mean duration from injury to surgery was 34.6 weeks (range, 24–43 weeks). The mean duration of the procedure was 143 min (range, 105–195 min). Nineteen patients achieved fracture healing and their mean follow-up period was 31.3 months (range, 24–120 months). No patients had obvious SL dissociation that was noted during the arthroscopy procedure. Nineteen patients achieved fracture healing, with a mean healing time of 16.3 weeks (range, 10–28 weeks). There were no perioperative complications. The 2 patients who developed nonunion after the index procedure were then lost to follow-up, and their last follow-up at around postoperative 1 year showed a VAS pain score of 5 during activity for both patients.

**Table 1:** Characteristics of the patients

Variables	Number or Values (range)
Patient number	21
Age (range)	31.8 years (19–55)
Duration from injury to surgery (range)	34.6 weeks (24–43)
Male/Female	6/15
Side of the hand (R/L)	14/7
Dominant hand	17
Nonunion location (Schernberg classification)	
I	2
II	19
Healing of the nonunion	19
Average healing time	16.3 weeks (10–28)
Follow-up duration	31.3 months (24–120)
Screw type (healed / total patients)	
Herbert	3/3
Headless Compression Screw (HCS)	13/15
Acutrak Standard	3/3
Screw removal in fracture-healed patients	17

Four patients were noted to have vascular compromise of the scaphoid in the MRI and obvious sclerotic density of the proximal fragment in the radiographs. The 2 patients that failed to heal were among these 4 patients.

In the final follow-ups with a mean of 31.3 months (range, 24–120 months), patients with fracture healing showed improvement in wrist flexion (from 54.5^o^ to 67.4^o^), wrist extension (from 62.3^o^ to 71.7^o^), DASH score (from 52.4 to 21.4), VAS during activity (from 4.6 to 2.1), and grip strength (from 9.6 kg to 24.7 kg), with statistical significance (Table 2), and were able to return to their previous activities with no or mild pain; no wrist braces were required.

**Table 2:** Characteristics and functional results of the 19 fracture-healed patients before surgery and at the final follow-ups

Case no.	Age (years)	Gender	Injury duration (weeks)	Healing time (weeks)	Follow- up duration (mos)	DASH		VAS during activity		Grip strength (kg)		Flexion (^o^)		Extension (^o^)		Pronation (^o^)		Supination (^o^)		Radial deviation (^o^)		Ulnar deviation (^o^)		Lateral SL angle (^o^)	
						B	A	B	A	B	A	B	A	B	A	B	A	B	A	B	A	B	A	B	A
**1**	29	F	35	13	24	50.0	12.5	4	2	11	30	75	78	70	80	80	80	80	80	10	15	30	28	69	65
**2**	36	F	32	25	36	46.7	25.0	6	2	7.5	21	48	60	54	62	75	75	80	80	18	17	29	26	95	75
**3**	26	F	42	24	24	51.7	19.2	5	3	12	24	57	65	55	65	80	85	85	90	11	20	25	27	60	60
**4**	52	M	38	10	24	57.5	29.2	3	1	10.5	22	46	60	68	75	70	70	70	75	12	20	28	25	79	67
**5**	23	F	30	19	24	48.3	21.7	5	2	12	29	50	65	50	75	85	85	85	90	12	18	25	29	85	64
**6**	55	M	40	17	24	54.2	33.3	6	3	8	20	58	75	63	75	70	70	80	80	17	16	30	27	84	71
**7**	38	M	27	18	30	45.8	24.2	3	1	10	22	55	70	66	70	75	75	85	80	25	16	27	27	98	61
**8**	25	F	25	14	27	55.0	25.8	5	3	12	24	65	70	65	70	80	85	85	85	23	16	26	26	88	74
**9**	47	F	37	28	24	58.3	27.5	5	2	7.5	20	60	60	76	85	70	70	80	85	17	19	30	26	89	65
**10**	27	F	32	10	24	55.8	12.5	6	3	8.5	24	60	65	62	61	70	75	85	85	13	22	28	28	97	68
**11**	28	F	24	11	27	52.5	22.5	4	2	11.5	27	58	75	72	75	80	80	85	85	22	19	27	28	74	68
**12**	32	M	39	19	24	53.3	10.8	5	1	9.5	24	44	57	61	80	75	80	85	85	15	15	26	25	90	53
**13**	29	F	43	16	24	45.8	18.3	3	1	9	20	52	75	72	75	75	75	85	85	12	15	28	30	99	62
**14**	40	F	41	20	25	53.3	30.0	6	4	8	22	51	60	58	65	75	75	85	85	11	25	26	27	76	62
**15**	22	F	33	12	25	58.3	19.2	6	3	8.5	22	43	65	53	65	80	85	85	90	14	18	30	30	78	63
**16**	23	F	40	14	24	59.2	24.2	5	2	11.5	23	59	75	67	75	75	80	85	85	21	23	25	25	87	70
**17**	20	M	36	16	120	57.5	10.8	4	2	9	36	55	70	55	70	85	85	85	90	16	24	29	30	70	65
**18**	19	F	36	12	40	48.3	20.0	3	1	6.5	25	50	70	51	70	85	85	85	90	15	22	28	30	65	65
**19**	33	M	28	12	24	45.0	11.7	4	1	10	34	50	65	65	70	80	80	85	85	15	23	28	28	75	68
Mean	31.8	–	34.6	16.3	31.3	52.4	21.4	4.6	2.1	9.6	24.7	54.5	67.4	62.3	71.7	76.5	78.7	82.8	84.7	15.7	19.1	27.6	27.5	82	66
*P* value	–	–	–	–	–	< 0.001		< 0.001		< 0.001		< 0.001		< 0.001		0.010		0.030		0.022		0.746		< 0.001	

B, before operation; A, at final follow-up after operation

The lateral SL angle was statistically significantly improved from a mean of 82° (range, 60^o^ to 99^o^) preoperatively, to 66^o^ (range, 53^o^ to 75^o^) postoperatively (*p* <  0.001). Seventeen patients requested screw removal after fracture healing. Most screw removal surgeries were performed 6–12 months after the index procedure under general anesthesia. After screw removal, a removable wrist orthosis was used for patient comfort, but was not routinely applied. Patients were allowed return to their previous work/activities one month after screw removal. The mean SL gap of the 17 patients was 2.2 mm at the time of screw removal, and 2.3 mm in the final follow-up, with a mean interval of 23.6 months (range, 12–112 months). The difference was not significant.

## Discussion

Arthroscopic-assisted bone grafting and SL screw transfixation for the treatment of proximal scaphoid nonunion yielded fracture healing in 19 of 21 patients (90.5%) in this study. These results are encouraging when compared to those of the open bone-grafting method for proximal scaphoid nonunion, in which fracture healing ranged from 90 to 100% [14, 25, 26]. Also, the healing time for our patients, at a mean of 16.3 weeks, was acceptable compared to that for proximal nonunion after open autologous bone grafting, which was reported to be 11.5–17.7 weeks [14, 15, 27].

There could be some intraarticular pathologies coexisting with a scaphoid nonunion. It has been reported in the literature that this constellation is perhaps not uncommon, and if present, the severity of the ligamentous injury is low [6, 28, 29]. With healing rates comparable to open methods, arthroscopic management has the advantages of providing a thorough wrist assessment, a comprehensive approach to address coexisting intraarticular pathologies, and minimal surgical trauma to the structure and vascularity, which is favorable for fracture healing [28, 30].

In treating chronic SL dissociation, RASL could be a treatment option [9, 18]. But in cases of scaphoid nonunion, the SLIL injury would most likely be minor. In our series, we did not see cases with obvious SL dissociation. Also, we did not denudate the SL junctional cartilage, as in the proposed RASL method [17, 18], but performed SL transfixation only. In the follow-ups of the 17 screw-removed patients, we found no further SL separation. So, it seems that screw fixation across the SL joint in proximal scaphoid nonunion treatment would not be harmful to the SL joint. But, as to the benefits for a probable concomitant SL injury after SL fixation, we still cannot draw a clear conclusion.

Rancy et al. reported that the proximal pole of the scaphoid would very likely have impaired vascularity in a fracture nonunion condition, but proximal scaphoid infarction is decidedly rare [27]. The key surgical method is to perform a thorough debriding of the nonunion site and non-vascularized autologous bone grafting. Rancy et al. reported an overall healing rate of 97.1% in treating 35 scaphoid nonunions. However, some surgeons prefer the use of vascularized bone graft, due to the accepted advantages of improved circulation and an enhanced bone healing process [10, 11, 31].

In our study, 2 of the 4 patients who had both vascular compromise of the scaphoid in the MRI and obvious sclerotic density in the radiographs failed to heal. This failure rate is considerable when using this arthroscopy-assisted method for proximal scaphoid nonunion with obvious vascular compromise, although the case number was small. But, in our opinion, if surgeons are familiar with arthroscopic treatment, this method is a minimally invasive surgery that is still worthy of being performed in this patient group, and would not hinder further surgeries too much, even if it fails. However, detailed communication with the patient prior to surgery is necessary and important.

Use of the exaggerated dumbbell-shaped screw in the RASL procedure is suggested (Fig. 2), since a thin part of the screw shaft located at the SL junction seems able to prevent screw loosening; however, the evidence is still inconclusive [17, 18]. We used conical screws in only 3 cases, and they all had fracture healing (Fig. 3). Because of the small number of cases, we cannot conclude that a conical screw could achieve healing success similar to that of a dumbbell-shaped Herbert-like screw for SL transfixation in proximal scaphoid nonunion treatment.

SL screw removal is not routinely suggested if there is no loosening [32]. However, the majority of our patients requested SL screw removal, and this was performed at least 6 months after fixation. This is because, in our culture, people prefer not to leave hardware in the body, if possible. Although we did not encounter complications with screw removal, we do agree that screw removal should be performed only when there is loosening or symptoms related to the screw.

We believe that the use of K-wires instead of the headless screw for SL transfixation would also be a good choice. In our results, the mean fracture healing time was 16.3 weeks. Thus, the fracture healing period with this arthroscopic treatment would not be too long. In addition, K-wires are easier to remove. As their diameter is small, K-wires could possibly be placed with less morbidity than a larger-sized headless screw. However, K-wires could possibly cause skin irritation.

Radial styloidectomy is an acceptable method for treating stage 1 SNAC, but we performed it in only 1 case (case 13). Whether radial styloidectomy will interfere with the results is still unclear. But, according to the study by Vutescu et al., radial styloidectomy should be performed with caution in cases with proximal pole scaphoid nonunion. Their results showed removal of part of the degenerated radial styloid could endanger the carpal extrinsic ligaments and cause carpal instability [33].

There were some limitations in this study, including its retrospective nature and the small number of cases. The majority of enrolled cases were female, which is not consistent with the reported epidemiology [34, 35]. This is because in the early enrollment period, male patients were still mainly treated with either the open vascularized or non-vascularized bone grafting method. Young female patients would often request treatment due to cosmetic concerns, so the arthroscopic method was performed more with them. Fracture healing was evaluated mainly with radiographs, which are not as precise as a CT scan. Also, the differences in patient characteristics and treatment, such as the use of different types of screws, the differences in the length of follow-up, and operations performed by 2 different surgeons, could also bias this study. The majority of our patients had screw removal, and this would be a drawback of the technique, in that the results would mainly show that the technique included temporary screw fixation across the SL joint for proximal pole scaphoid nonunion.

## Conclusions

For proximal scaphoid nonunion, arthroscopic-assisted reduction, bone grafting and screw fixation across the SL joint could be an alternative treatment if the surgeon is familiar with arthroscopic procedures. A good healing rate and functional outcomes could be achieved. However, further comparative studies are still needed.

## Data Availability

The datasets used and/or analyzed during the current study are available from the corresponding author on reasonable request.

## Abbreviations

ARASL: Arthroscopic reduction-association of the scapholunate
DASH: the Disabilities of the Arm, Shoulder, and Hand
DISI: Dorsal intercalated segmental instability
HCS: Headless Compression Screw
MRI: Magnetic resonance imaging
SNAC: Scaphoid nonunion advanced collapse
SL: Scapholunate
SLIL: Scapholunate interosseous ligament
VAS: Visual Analog Scale
